# *In vitro* ruminal fermentation, methane emissions, and nutritional value of different tropical feedstuffs for ruminants

**DOI:** 10.5455/javar.2024.k842

**Published:** 2024-12-27

**Authors:** Muhammad Khairul Bashar, Eva Haese, Nasrin Sultana, Markus Rodehutscord

**Affiliations:** 1 Department of Animal Nutrition, Institute of Animal Science, University of Hohenheim, Stuttgart, Germany; 2 Department of Animal Nutrition, Institute of Animal Science, University of Bonn, Bonn, Germany; 3 Bangladesh Livestock Research Institute, Savar, Bangladesh

**Keywords:** *In vitro* digestibility, nutritional composition, methane emissions, tropical feedstuffs

## Abstract

**Objective:**

This research aimed to evaluate *in vitro* ruminal fermentation, methane (CH_4_) emissions, and the relationship between the nutritional content and CH_4_ emissions of tropical feedstuffs to formulate low CH_4_-emitting feeds for ruminants.

**Materials and Methods:**

Eighteen feedstuffs, including roughages (3 crop residues, 2 silages, 3 common grasses, and 4 leguminous fodder) and 6 concentrates, were evaluated using the Hohenheim Gas Test. Approximately 200 mg of feed were incubated with a rumen fluid-buffer solution for 72 h to test gas production (GP) and 120 mg for 24 h to determine the CH_4_ concentration in the gas. Digestibility of organic matter (dOM) and metabolizable energy (ME) were calculated using GP data.

**Results:**

Leguminous fodder contained the highest crude protein (CP) concentration (166–314 gm/kg dry matter (DM)), followed by common grasses (52–147 gm/kg DM) and silages (94–106 gm/kg DM), but the lowest concentration of detergent fiber fractions. Crushed wheat and maize had higher dOM and ME (87.8% and 90.9%, and 14.4 MJ/kg DM and 13.8 MJ/kg DM, respectively), and their CH_4_ concentration (% of GP) and CH_4_ emissions (L CH_4_/kg dOM) followed a similar trend as the other feedstuffs. The dOM and ME of German grass and Ipil-ipil were higher, whereas the CH_4_ concentration and CH_4_ emissions were lower compared to crop residues and other common grasses. The CH_4 _emissions originating from the feedstuffs were positively correlated with the concentration of neutral detergent fiber and GP and negatively correlated with CP.

**Conclusion:**

Our result provides an opportunity to select feed ingredients with higher digestibility and concurrently less CH_4_ emissions in formulating diets for ruminants when using commonly available feed resources in many tropical countries. This may enhance animal productive performances while reducing the impact of animal production on the environment.

## Introduction

The increased global population demands more food, and a significant part, particularly protein, comes from livestock, which can have negative effects on the environment. To adequately satisfy the nutritional requirements of ruminants while simultaneously mitigating environmental burdens, such as greenhouse gas (GHG) emissions, it is crucial to enhance the accessibility of feed resources. In tropical countries, ruminant production systems are often associated with low feed efficiency and high emission intensities [1] because of inadequate nutrition [2,3,4], including low nutrient digestibility [5]. Consequently, the ruminal fermentation of the feed is impacted, with an increase in digesta retention time and a decrease in rumen absorption of volatile fatty acids (VFAs) reducing cattle production efficiency. As a result, there is a higher rate of methane (CH_4_) emission per kg of milk and meat obtained [6].

A major challenge for tropical countries, such as Bangladesh, is increasing production without further impacting the environment by the livestock sector. Better characterization of the feeding value of tropical feedstuffs and related features of potential CH_4_ emissions is required to formulate mixed rations needed to increase animal productivity [7,8,9]. To the best of our knowledge, the digestibility of organic matter (dOM), metabolizable energy (ME), and CH_4_ emissions of commonly used feedstuffs available in Bangladesh and used in the diet of ruminants have not been determined. The dOM and ruminal fermentation are related to CH_4_ emissions [10]. *In vitro* gas production (GP) techniques, such as the Hohenheim Gas Test (HGT) reported by Menke and Steingass [11], can be used to analyze CH_4_ emissions of these commonly used feedstuffs. These procedures are relatively cheap and widely used for feed evaluation, including tropical feeds [12,13]. In contrast, *in vivo* procedures, although reliable for comprehensive evaluation, are expensive, laborious, and time-consuming [14]. In Bangladesh and other tropical countries alike, there is a need for more data on the potential CH_4_ emissions of common tropical feedstuffs and how they relate to the nutrient components of these feeds. Therefore, this study aimed to generate data on nutritive characteristics, digestibility, and CH_4_ emission of most commonly used ruminants’ feedstuffs so that less CH_4_-emitting diets of ruminants could be formulated to mitigate GHG emissions from ruminants in Bangladesh and similar other tropical countries. The main objectives of the study were: i) to characterize commonly used feedstuffs chemical composition; ii) to assess the* in vitro* GP kinetics and CH_4_ emissions; and iii) to relate CH_4_ emissions to the chemical composition and *in vitro* GP attributes of the feedstuffs.

## Materials and Methods

### Ethics approval

The rumen-cannulated cows used as donor animals of inoculum for *in vitro* incubation were housed at the Agricultural Experiment Station of Hohenheim University at Meiereihof in Stuttgart-Hohenheim (Germany), in strict accordance with the German Animal Welfare legislation. All procedures regarding animal handling were approved by the Regierungspräsidium Stuttgart, Germany (approval code V352/18 TE).

### Sample collection and preparation

Eighteen feedstuffs, 12 roughages, and 6 concentrate ingredients commonly used in Bangladesh for ruminant feeding were selected to be evaluated in this study. These are: a) crop residues such as rice straw (*Oryza sativa*), urea-molasses-treated straw (UMS), and maize stover (*Zea maize*); silages like maize silage (*Zea maize*) and Napier silage (*Pennisetum purpureun*); common grasses like German grass (*Echinochloa polystachya*), para grass (*Brachiara mutica*), and Napier grass; leguminous fodder such as Ipil-ipil (*Leucaena leucocephala*), Gliricidia (*Gliricidia sepium*), alfalfa hay (*Medicago sativa*), and moringa tops (*Moringa oleifera*), and b) concentrate ingredients such as crushed maize (*Zea mays*), crushed wheat (*Triticum aestivum*), wheat bran, khesari bran (*Lathyrus sativus*), rice bran (*Oryza* *sativa*), and mustard oil cake (*Brassica juncae Coss*) were collected from Bangladesh Livestock Research Institute (BLRI), Savar, Dhaka1341 Bangladesh. The feedstuffs collection and preparation procedure were the following:

**Table:** 

Feedstuffs	Collection and preparation procedure
Rice straw	After harvesting the grain, the straw was sun-dried on an open field for 3–4 consecutive days, then chopped using an electric chopper (Borna electronic, 65/C Shatmatha, Bogra, Bangladesh) to achieve particles with a length of 5–6 cm.
UMS	Urea molasses rice straw (UMS) was prepared as a mix: 10 kg dry chopped rice straw, 5 kg water, 1.7 kg molasses, 300 gm urea.
Maize stover	Residue of maize plant grown for grain production that includes stalks, leaves, and husks. After harvesting, it was cut as described for the rice straw.
Maize silage	After harvesting maize plants at the milk stage, it was cut using the chopper to achieve particle lengths of 6–8 cm and kept in a silo pit for 30 days following the BLRI practices of pit silage preparation [15,16].
Napier silage	For silage preparation, 45–50-day-old Napier grass was harvested from 5 to 6 cm above the ground, chopped into a length of 6–8 cm, and ensiled in a silo for 30 days.
Common grasses	The common grasses (Napier, German, and para grass) were cultivated following the standard agronomical practices [15] recommended by the BLRI. At 45–50-day intervals, the grasses were harvested and cut to 5–6 cm.
Leguminous fodder	Every 65–75 days, the leaves with twinges of Ipil-ipil, *Gliricidia* and moringa tops were harvested and sun-dried on a smooth concrete floor with a polythene sheet for two consecutive days. Material was transferred into the polythene bags, sealed, and stored.

All feedstuffs were oven-dried at 63°C for 48 h and ground through a 2.0-mm sieve (Retsch GmbH, 5657 HAAN, Germany). After the arrival of samples at the University of Hohenheim, all feedstuffs were ground again using a Wiley Mill (Dietz-Motoren, KG, Elektromotorenfabrik, 7311 Dettinger-Tech, Germany) screened with a 1.0-mm mesh sieve to be used in the HGT and a 0.5-mm mesh sieve for chemical analysis. All ground materials were stored at ambient temperature in plastic bottles until further use.

### Nutrient analysis

Analysis of chemical fractions was performed following the official methods in Germany [Verband Deutscher Landwirtschaftlicher Untersuchungs- und Forschungsanstalten (VDLUFA) 2007]. All samples of dry matter (DM) were determined by oven-drying for 4 h at 103°C (method 3.1), followed by combustion at 550°C for 4 h to measure the crude ash (CA) (method 8.1). To determine the crude protein (CP) concentration, the nitrogen (N) concentration was determined by the Kjeldahl method, comprising acid digestion of samples with sulfuric acid (H_2_SO_4_), steam distillation, and determination of ammonium formed by titration, and multiplied by 6.25 (method 4.1.1). The neutral detergent fiber (NDF) of samples was determined by pre-treating samples with heat-stable amylase (aNDF) (method 6.5.1), followed by acid detergent fiber (ADF) (method 6.5.2), and acid detergent lignin (ADL) (method 6.5.3). A bomb calorimeter (C 200; IKa-Werke GmbH and Co. KG Staufen, Germany) was used to assess the gross energy (GE) applying benzoic acid as a standard. All determinations were made in duplicate.

### Animals and diet

Fresh rumen fluid was collected from two ruminally fistulated Jersey cows that were fed a total mixed ration (TMR). The TMR consisted of 32% maize silage, 23% grass silage, 20% concentrate mixture (17% maize, 20% soybean meal, 25% barley grain, 28% wheat grain, 4% molasses, and 6% vitamin-mineral premix), 10% meadow hay, 2% barley straw, 1% mineral mixture, and 12% water. The cows had *ad libitum access to* feed and drinking water. Care and use of the animals were governed by German animal welfare legislation and approved by the Regierungspräsidium Stuttgart, Germany (approval code V352/18 TE).

### In vitro GP kinetics

The 1^st^ incubation was performed to determine the* in vitro* GP kinetics using the HGT as described by Menke and Steingass [11]. Briefly, approximately 200 ± 5 mg of DM of feed samples was weighed and placed into the bottom of 100-ml syringes, ensuring that the sample did not adhere to the wall of the syringe. Then, the syringes were closed airtight with greased plungers and pre-warmed (39°C) in an incubator. The incubation medium, without rumen fluid, was prepared the day before the study day using distilled water, 620 ml; micro-mineral solution, 160 µl; buffer solution, 310 ml; macro-mineral solution, 310 ml; and resazurin solution, 1,600 µl volume for all syringes (46). It was kept in a water bath maintained at 39°C under continuous stirring with a magnetic stirrer and CO_2_ flow. On the study day, the reduction solution (distilled water, 62 ml; sodium hydroxide (NaOH), 2.6 ml; and sodium sulfide (Na_2_S × 7H_2_O), 373–400 mg for 46 syringes) was freshly prepared and added to the incubation medium. Rumen fluid was obtained on the study day before the morning feeding, mixed from both cows at a 1:1 ratio, filtered through a double cheesecloth layer, and 650 ml was added to the solution. After 15 min, the rumen fluid with the incubation medium was pumped into the syringe (30 ml) and carefully handled to ensure the absence of air bubbles on the syringe surface and placed into the incubator for 72 h at 39°C. Five experimental runs were conducted, where each run included 18 samples (12 roughages and 6 concentrates) with two replicates per sample (total replication of 10 per sample). Furthermore, each run had 46 syringes, with 36 syringes containing an experimental feed sample (18 × 2 = 36), 4 syringes for a blank, 3 syringes for hay, and 3 syringes for concentrate standard. The arrangement of the syringes in the oven was randomized. The cumulative GP was recorded after 2, 4, 6, 8, 12, 24, 32, 48, and 72 h of incubation. The mean GP for standards and blanks was used to correct the GP of the feed samples at each incubation time.

The following non-linear regression was fitted to the GP data according to Seifried et al. [17]:

Y =* p*GP (1-e^-*c*GP 0.01t^)

where GP is the GP after *t* h of incubation (ml/200 mg DM), *p*GP is the potential GP (ml/200 mg DM), *c*GP is the GP rate constant (%/h), and *t* is the incubation time (h).

The dOM and ME were estimated using the GP value obtained after 24 h and analyzed nutrients using the equations 12f and 43f by Menke and Steingaß [11]:

dOM (%) = 14.88 + 0.8893 GP_24_ + 0.0448 CP + 0.0651 CA

ME (MJ/kg DM) = 1.24 + 0.1457 GP_24_ + 0.0070 CP + 0.0224 CF

where GP_24_ is GP within 24 h of incubation (ml/200 mg DM), CP is crude protein (gm/kg DM), CA is crude ash (gm/kg DM), and CF is crude fat (gm/kg DM).

### CH_4_ emissions and CH_4_ concentration

Following the first incubation, the second incubation was carried out to quantify CH_4_ emission. The incubation included 120 mg of DM feed sample for 24 h using the same approach as in the initial incubation. For this incubation, four experimental runs were conducted, including all feedstuffs (12 roughages and 6 concentrates), with two replications per run for each feed. In addition, each run also contained four blank syringes, which contained only buffer solution without feed (called blank), three syringes with hay, and three syringes containing concentrate standard sample.

After 24 h of incubation, the GP was recorded and the CH_4_ concentration of the gas was measured using an infrared- CH_4_ analyzer (Pronov Analysentechnik GmbH Co. KG, Berlin, Germany) calibrated with a reference gas (13.0 vol% CH_4_; Westfalen AG, Münster, Germany). The produced CH_4_ volume (ml) was calculated by multiplying the GP (ml) by CH_4_ concentration (%) divided by 100 and standardized to 120 mg DM of feed. The CH_4_ and GP values were corrected for the data obtained for the blanks. The CH_4_ concentration (%) of the produced gas after correction of blank values was then calculated as the CH_4_ volume (ml/120 mg DM) divided by the total GP (ml/120 mg DM) and multiplied by 100.

### Statistical analysis

The Pearson correlation coefficients between CH_4_ emissions and other variables were calculated. Stepwise multiple regression equations were calculated using aNDF, ADF, ADL, CP, and GP_24_ as the input variables and 24 h CH_4_ emissions as the output variable (72 observations from 12 feedstuffs with 6 replications).

## Results

### Nutrient composition and gross energy of feedstuffs

Among the roughages, CP concentration was the highest in leguminous fodder (166–314 gm/kg DM), followed by common grasses (52–147 gm/kg DM) and silages (94–106 gm/kg DM), while concentrations of ADF, aNDF, and ADL were the lowest in leguminous fodder (Table 1). Ipil-ipil contained the highest concentration of CP and the lowest concentrations of aNDF, ADF, and ADL. Crop residues contained the lowest CP concentration (44–70 gm/kg DM) and the highest concentrations of fiber fractions. The crude ash (CA) concentration was the highest in German grass and the lowest in maize stover. The GE concentration of leguminous fodder was higher than that of crop residues, common grass, and concentrates. Among the concentrates, khesari bran contained the highest CP and the lowest ADL (Table 1).

**Table 1.** Analysed nutrient composition and gross energy of the tested feedstuffs.

Feedstuff group	Name of feed	DM (%)	Chemical fractions (gm/kg DM)							GE (MJ/kg DM)
			CA	CL	CP	CF	aNDF	ADF	ADL	
Roughages	Crop residues									
	Rice straw	93.7	136	6.0	44.3	190	743	547	41.6	16.9
	UMS	94.1	141	5.9	69.7	136	580	424	28.6	16.7
	Maize stover	94.7	64.2	8.1	54.1	146	682	432	44.6	18.6
	Silage									
	Maize silage	95.7	80.9	8.5	107	159	712	484	54.3	18.9
	Napier silage	96.1	71.0	8.3	93.7	206	771	559	84.3	18.8
	Common grasses									
	Napier grass	95.2	87.9	10.2	52.2	188	721	528	75.5	18.3
	German grass	94.6	155	11.3	147	163	626	485	67.6	17.4
	Para grass	96.1	90.7	8.7	67.4	173	695	486	77.1	16.6
	Leguminous fodder									
	Gliricidia	90.7	117	24.7	239	140	524	500	301	20.2
	Ipil-ipil	92.7	92.1	38.4	314	70.3	380	187	74.2	21.1
	Alfalfa hay	93.1	106	14.0	166	189	586	536	122	18.8
	Moringa tops	95.7	66.0	14.0	125	175	563	497	83.4	19.1
Concentrates	Crushed maize	91.3	23.3	20.9	83.9	5.4	86.4	34.5	10.9	19.7
	Crushed wheat	91.5	15.7	62.2	118	8.1	121	40.2	9.10	18.8
	Wheat bran	90.9	41.6	18.7	131	39.8	276	167	44.3	18.7
	Khesari bran	90.9	111	25.4	163	95.8	409	312	38.4	17.3
	Rice bran	93.5	143	47.3	75.1	188	636	533	144	19.5
	M. oil cake	94.5	389	83.6	104	54.7	462	363	57.2	13.3

UMS = Urea molasses treated rice straw, M. oil cake = Mustard oil cake, DM = Dry matter, CA = Crude ash, CL = Crude fat, CP = Crude protein, CF = Crude fiber, aNDF = Neutral detergent fiber, ADF = Acid detergent fiber, ADL = Acid detergent lignin, GE = Gross energy.

### In vitro ruminal fermentation characteristics and nutritive values

Within roughages, crop residues of maize stover, German grass, and Napier grass produced higher gas volumes at 24 h, while the lowest was observed in *Gliricidia *(Table 2). The highest *pGP *among the roughages was calculated for rice straw (60.0 ml/200 mg DM), greater than that of all other roughages. The leguminous fodder of *Moringa* tops had the highest *c*GP among all roughages, which was 12.1%/h. Among the concentrates, the crushed maize and crushed wheat showed the highest GP_24_ and *p*GP, whereas the lowest GP_24_ and *p*GP were obtained for rice bran.

The calculated values of dOM and ME of the feedstuffs are shown in Table 3. All roughages had different dOM and ME, with German grass and Ipil-ipil exerting the highest and rice straw the lowest values. The dOM and ME of crushed maize and crushed wheat were higher than those of other concentrates. Rice bran showed the lowest values among all tested feedstuffs.

**Figure 1.**
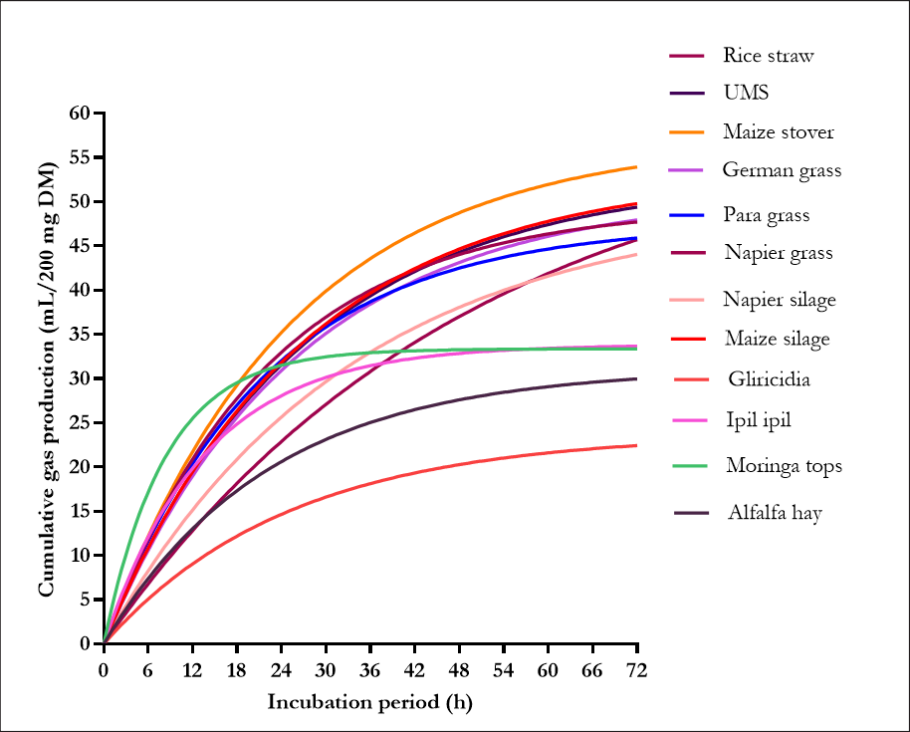
Cumulative gas production profile of the roughages. *In vitro* gas production profile has been fitted to curves using the equaliton Y = *p*GP (1-e^-*c*GP 0.01t^). The estimated parameters of the nonlinear functions are presented in Table 2.

**Figure 2.**
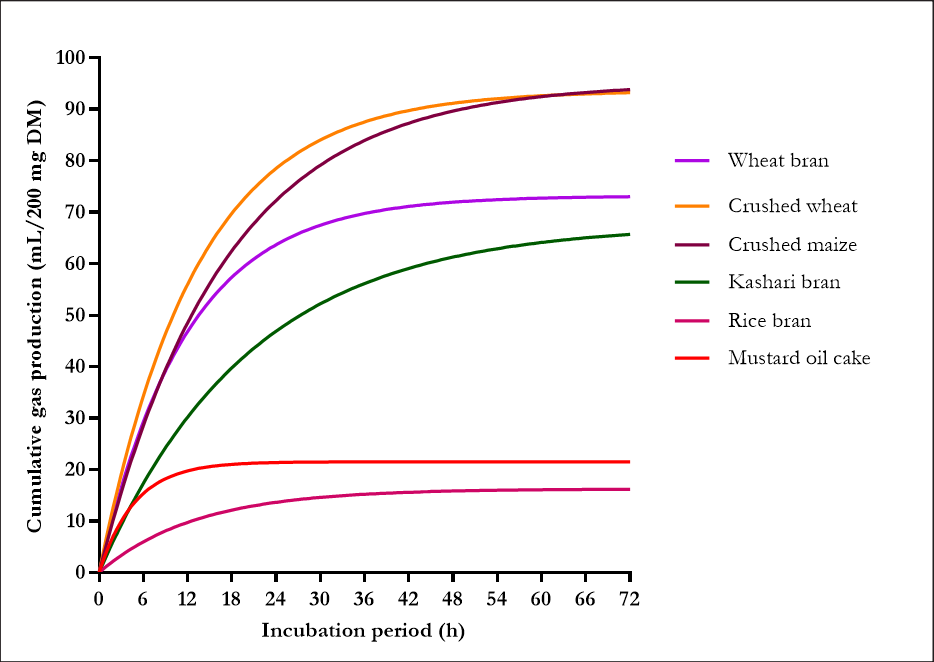
Cumulative gas production profile of the concentrates. *In vitro* gas production profile has been fitted to curves using the equaliton Y = *p*GP (1-e^-*c*GP 0.01t^). The estimated parameters of the nonlinear functions are presented in Table 2.

Figures 1 and 2 show the GP patterns of the *in vitro* fermentation of roughages and concentrates, respectively. The total volume and pattern of GP varied among all feedstuffs; however, the observed differences were not consistent for the different incubation periods, except for crushed wheat and crushed maize, which consistently produced the highest volume of gas across all incubation times. The lowest GP was measured for *Gliricidia* and rice bran from 8 to 72 h compared to all other feedstuffs.

### In vitro CH_4_ emissions and related traits

The values of CH_4_ emissions after incubation for 24 h are shown in Table 4. The values of CH_4_ emissions, CH_4_ concentration, and CH_4_/dOM in rice bran were the lowest, while the loss of energy in the form of CH_4_ was the highest compared to all other feedstuffs. *Gliricidia* produced the lowest CH_4_ volume, but the CH_4_ concentration and CH_4_ energy loss were the highest among all roughages. The CH_4_ concentrations in the total gas of maize silage and Ipil-ipil were lower than those of the other roughages. However, the wheat bran produced the lowest values among all feedstuffs. The opposite trend was recorded in crushed wheat, crushed maize, and khesari bran, which produced the highest concentrations of CH_4_ and CH_4_/dOM; nevertheless, the energy loss as CH_4_ was very low (1.40%, 1.92%, and 2.34% of the GE).

**Table 2.** *In vitro *gas production kinetics of the tested feedstuffs.

Feed-stuff group	Name of feed	GP_24_ (ml/200 mg DM)	*p*GP (ml/200 mg DM)	*c*GP (%/h)
Roughages	Crop residues			
	Rice straw	25.0	60.0	2.02
	UMS	31.1	53.0	3.80
	Maize stover	34.0	57.2	4.00
	Silage			
	Maize silage	32.5	53.3	3.80
	Napier silage	27.8	49.8	3.03
	Common grasses			
	German grass	33.7	51.1	3.88
	Para grass	32.0	47.6	4.68
	Napier grass	32.8	49.6	4.58
	Leguminous fodder			
	Ipil ipil	27.0	34.0	7.39
	Gliricidia	15.2	23.8	3.98
	Alfalfa hay	21.2	31.1	4.55
	Moringa tops	29.5	33.4	12.1
Concentrates	Crushed maize	78.9	95.3	5.94
	Crushed wheat	74.9	93.6	7.60
	Wheat bran	60.6	73.4	8.50
	Khesari bran	47.3	67.7	4.93
	Rice bran	13.5	16.3	7.65
	M. oil cake	21.5	21.5	21.3

M. oil cake = Mustard oil cake, GP_24_ = Gas production at 24 h, *p*GP = Potential gas production, *c*GP = Gas production rate constant.

**Table 3.** *In vitro* dOM matter and metabolisable energy of the tested feedstuffs.

Feedstuff group	Name of feed	dOM (%)	ME (MJ/kg DM)
Roughages	Crop residues		
	Rice straw	47.9	5.32
	UMS	54.9	6.39
	Maize stover	51.6	6.73
	Silage		
	Maize silage	53.8	6.91
	Napier silage	48.5	6.13
	Common grasses		
	German grass	61.6	7.43
	Para grass	52.3	6.57
	Napier grass	52.1	6.60
	Leguminous fodder		
	Ipil ipil	58.9	8.22
	Gliricidia	46.8	5.69
	Alfalfa hay	48.1	5.80
	Moringa tops	50.9	6.72
Concentrates	Crushed maize	90.3	13.8
	Crushed wheat	87.8	14.4
	Wheat bran	77.3	11.4
	Khesari bran	71.5	9.84
	Rice bran	39.5	4.78
	Mustard oil cake	64.1	6.98

dOM = Digestibility of organic matter, ME = Metabolisable energy.

### Associations between CH_4_ emissions, chemical constituents, and fermentation characteristics

Across all feedstuffs studied CH_4_ emissions were negatively correlated with the CP concentration (−0.86, *p <* 0.01) (Table 5). A significant positive correlation was observed between CH_4_ emissions and the aNDF concentration (0.67, *p <* 0.05) and GP_24_ (0.94, *p <* 0.05). The concentrations of other nutrients were not significantly correlated with CH_4_ emissions.

Linear regression equations were derived to predict CH_4_ emissions from the chemical constituents and *in vitro* ruminal GP, as shown in Table 6. The fiber fractions alone, including aNDF, ADF, and ADL, were poor indicators of CH_4_ emissions (*R*^2^ = 0.57) in the linear regression (*p <* 0.06) for all feedstuffs. The consideration of CP and aNDF concentrations as predictors of CH_4_ emissions increased the prediction accuracy (*p <* 0.01). The CP concentration alone was a good predictor of CH_4_ emissions (*R*^2^ = 0.72, *p <* 0.01). GP_24_ alone showed a positive relationship with CH_4_ (*R*^2^ = 0.89, *p <* 0.01).

## Discussion

### Chemical composition of all feedstuffs

Most feedstuffs particularly crop residues and common grasses used in this study were deficient in CP (44.3–69.7 gm/kg DM and 52.2–147 gm/kg DM, respectively) but contained high cell wall content (ADL; 424–547 gm/kg DM and 485–528 gm/kg DM, aNDF; 580–743 gm/kg DM and 625–721 gm/kg DM, respectively). However, the CP content of the roughage such as Ipil-ipil used in the present study was 314 gm/kg suggesting there is a great opportunity to meet the protein requirement of ruminants using Ipil-ipil. A sufficient quantity of dietary CP, particularly rumen-degradable protein, is essential for maintaining microbial protein production, a component of metabolizable protein necessary for the host [18]. Our results indicate Ipil-ipil could be used in the diet of ruminants in the tropics to meet their dietary requirements. A threshold value of 7.0% CP has been suggested as acceptable forage quality [19], below which microbial fermentation of roughages may be limited due to a shortage of nitrogen, and host animals may be undersupplied with metabolizable protein [19].

**Table 4.** CH_4_ emissions of the tested feedstuffs.

Feedstuff group	Name of feed	CH_4_ (ml/120 mg DM)	CH_4_ conc. in GP (%)	L CH_4_/kg dOM	CH_4_ energy (% GE)
Roughages	Crop residue				
	Rice straw	2.79	15.9	47.3	4.32
	UMS	3.31	16.3	49.7	3.63
	Maize stover	3.43	15.6	54.8	3.50
	Silages				
	Maize silage	2.95	14.3	44.2	4.08
	Napier silage	2.70	14.6	44.6	4.46
	Common grass				
	German grass	3.23	15.1	42.9	3.73
	Para grass	3.13	15.6	49.3	3.84
	Napier grass	3.29	15.9	51.0	3.65
	Leguminous fodder				
	Ipil ipil	2.46	14.7	34.8	4.86
	Gliricidia	1.62	18.2	28.8	7.41
	Alfalfa hay	2.41	17.3	41.2	4.97
	Moriga tops	2.82	15.9	43.6	4.29
Concentrates	Crushed maize	6.36	14.8	58.2	1.92
	Crushed wheat	8.54	18.3	81.1	1.40
	Khesari bran	6.72	18.7	58.9	2.34
	M. oil cake	5.12	17.5	26.5	5.73
	Rice bran	0.94	13.5	18.7	13.1
	Wheat bran	2.11	17.3	73.2	1.78

CH_4_ = Methane, CH_4_ conc. = Methane concentration, GP = Gas production at 24 h, dOM = Digestibility of organic matter DM = Dry matter, GE = Gross energy.

**Table 5.** Pearson correlation between *in vitro* CH_4_ emissions (ml/120 mg DM), chemical constituents, and gas production.

	CH_4_	Nitrogen and gas production	CH_4_
Crude ash (CA)	0.04	Crude protein (CP)	-0.86**
Neutral detergent fiber (aNDF)	0.67*	Gas production (GP) at 24 h	0.94*
Acid detergent fiber (ADF)	0.19		
Acid detergent lignin (ADL)	-0.36		

CH_4_ = Methane, NS = Not significant. *p < 0.05; **p < 0.01.

In the present study, most of the roughages had a CP level higher than the threshold of 7.0%, except crop residues, Napier silages, and common grasses. The high CP content of Ipil-ipil (314 gm/kg DM) and *Gliricidia* (240 gm/kg DM) suggests that these feeds can be used as a supplement for ruminants where local roughages are deficient in CP [20,21]. Rice straw’s CP content of 44 gm/kg DM was below the threshold; however, crop residues likely still make up a significant portion of ruminant feeds in many parts of the world.

Roughages with an NDF content below 35.5% are considered to be of high quality, while those with an NDF level above 46.0% are deemed to be of low quality [ 22]. Only Ipil-ipil exhibited an NDF value around the threshold in the present study. The ADF concentration was also low (187 gm/kg DM), which can have a favorable effect on roughage quality because low ADF concentration indicates greater digestion of the feed. Compared to other feed ingredients, Ipil-ipil had the lowest NDF and ADF concentrations, while Napier silage and rice straw had the highest. This low fiber in association with the high CP of Ipil-ipil indicates Ipil-ipil forage could be used for ruminant production in the tropics. The lowest ADL value was measured in UMS (28.6 gm/kg DM) and the highest in *Gliricidia *(301 gm/kg DM) (Table 1). Many factors may affect the cell wall fractions, such as the stage of maturity, variety or species, growth environment, and soil type [23,24]. The majority of the roughages used in this study had a high cell wall content, similar to crop residues; aNDF ranged from 58.0% to 74.3%, and GP ranged from 25.0 to 34.0 ml/200 mg DM at 24 h, which may have been inhibited associated with lower microbial activity by limiting the accessibility of readily fermentable carbohydrates. This can also cause higher energy loss due to increased CH_4_ emissions and reduced animal production efficiency [25].

**Table 6.** Linear regression equation to predict CH_4_ from chemical constitutes and GP_24_ across all feedstuffs.

Equations	*R^2^*	*p*- value
CH_4_ = 0.20 aNDF + 0.03ADF–0.66ADL + 14.0	0.57	< 0.06
CH_4_ = - 0.10 aNDF–0.56CP + 37.0	0.76	< 0.00
CH_4_ = - 0.44 CP + 29.1	0.72	< 0.00
CH_4_ = 0.25 aNDF + 7.65	0.45	< 0.02
CH_4_ = 0.70 GP_24_ + 3.70	0.89	< 0.001

CH_4_ = Methane (L/kg DM) , aNDF = Neutral detergent fiber (gm/kg DM), ADF = Acid detergent fiber (gm/kg DM), CP = Crude protein (gm/kg DM), GP_24_ = Gas production at 24 h (ml/200 mg DM).

### In vitro ruminal fermentation characteristics of feedstuffs

The concentrate feeds produced more gas with the rapid fermentation of available carbohydrates, which is a good indicator for extended digestion. Variations in the chemical components of the feedstuffs may account for differences in total gas and CH_4_ emissions. Total GP was highest after 24 h for all concentrates used in the present study except rice bran. Conversely, after 24 h, the average total GP of protein-rich leguminous and fibrous feeds was lower. Consistent with Singer et al. [26], it can be assumed that cereals had more readily fermentable carbohydrates and a higher degree of ruminal fermentation than fibrous, protein-rich legumes and forages. The higher dOM of cereals compared to the other categories of feedstuffs is a direct reflection of the exclusive ruminal fermentation that occurs due to their high starch and lower structural carbohydrate contents.

The leguminous fodder of Ipil-ipil and *Gliricidia* produced low amounts of gas at 24 h (27.0 ml/200 mg DM and 15.2 ml/200 mg DM, respectively). Protein-rich diets release amino acids, peptides, and ammonia by microbial protein degradation, eventually converted in parts to microbial proteins. Although dietary soluble proteins ferment rapidly, less gas is produced during protein fermentation than in carbohydrate fermentation [27]. Ipil-ipil also contained secondary plant metabolites such as tannins and saponins that may have impacted the volume of gas produced [28]. However, the presence of secondary plant constituents was not evaluated in the present study. Rice straw and other roughages contained a greater level of fiber fractions; however, the total GP of other roughages was markedly higher than that of rice straw. Starch, sugar, and NDF-containing feeds ferment faster than ADF-containing feeds; nevertheless, lignin cannot be decomposed by cellulolytic rumen microbes [27]. The other forages used in the present study had a lower lignin content, which likely led to a higher total GP and higher dOM than that achieved with rice straw.

The concentrate feeds were found to have the highest dOM and ME compared to the other feedstuffs. The crushed maize and wheat exhibited higher dOM and ME content. Feed that has a dOM higher than 50% has a good chance of delivering the ME necessary to support animal production.

### In vitro CH_4_ emissions and related traits

Anaerobic decomposition of cell walls containing slowly fermentable carbohydrates such as cellulose and hemicellulose in feedstuffs is associated with high *in vitro* CH_4_ emissions. The major methanogens in the rumen utilize H_2_ as a significant energy source to reduce CO_2_ to CH_4_. Therefore, CO_2_ and H_2_ are positively correlated with CH_4_ during microbial fermentation of feed in the rumen [29]. During the 24 h incubation period, the CH_4_ emissions (ml/120 mg DM) and its concentration (% of GP) from the examined feedstuffs varied substantially. The relatively high CH_4_ emissions of concentrates, except wheat bran and rice bran, could be attributed to the high amount of fermentable starch, sugars, or hemicelluloses as substrates for rumen microorganisms. These soluble carbohydrates enhance ciliate protozoa and accelerate their hydrogen transfer to methanogens, leading to a significant increase in CH_4_ emissions [30]. Rice bran has a high content of unsaturated fatty acids [31] that can be hydrogenated by rumen microorganisms. This process results in a decreased pressure of H_2_ and less demand for CH_4_ emissions. In addition, fat is thought to decrease CH_4_ formation by accelerating propionate production and inhibiting protozoa activity, as well as suppressing cellulolytic bacteria and feed digestion in the rumen, which may have happened in the current study.

The CH_4_ concentration in the produced gas can be measured to assess the ability to suppress CH_4_ release by *in vitro* methods [32]. A low CH_4_ concentration implies that a candidate would be a more effective rumen modulator for CH_4_ reduction than a high yield. Among the roughages examined in the present study, leguminous fodder like Ipil-ipil demonstrated the lowest concentration of CH_4_ (14.7%), indicating its potential as a species for effectively reducing CH_4_ emissions. Two factors may be responsible for this; the first is the high CP content, making it an ideal protein supplement source for ruminant feed, which may increase microbial protein synthesis. Furthermore, most of the high CP-containing leguminous fodder has a promising reduction potential for CH_4_ owing to the lower production of H_2_ and CO_2_ of protein than carbohydrates [33,34]. The second factor is that Ipil-ipil also contains secondary plant metabolites, such as tannins and saponins, which are of substantial interest for CH_4_ mitigation [35,36]. It also rationally modulates the rumen microbiome and modifies its function, reducing feed energy loss as CH_4_, which also increases microbial protein synthesis and fiber degradation in tropical feedstuffs [37,38,39].

### Associations between CH_4_ emissions, chemical constituents, and fermentation characteristics

Data generated in this study was also used in predicting CH_4_ emissions of feedstuffs based on the relationship between CH_4_ emissions, chemical ingredients, and fermentation characteristics as suggested by Santoso and Hariadi [40] and Navarro-Villa et al. [41]. Equations developed may offer the users the opportunity to calculate CH_4_ emissions of feed ingredients used to select feed in formulating diets that could reduce CH_4_ emissions as well as to compare data generated from future *in vivo* trials on ruminants.

In the present investigation, correlation analysis across feedstuffs revealed that feed ingredients with high CP had a negative influence on *in vitro* CH_4_ emissions. This result is consistent with that reported by Singh et al. [27], who indicated that increasing protein and non-degradable cell wall fractions would decrease *in vitro* CH_4_ emission. In contrast, increasing GP and dOM were positively linked with CH_4_ emissions because feedstuffs contained more fermentable substrates [41]. A significant connection was calculated between CH_4_ emissions and aNDF concentration for all feedstuffs, suggesting that an increase in the content of degradable cell-wall fractions is responsible for the higher CH_4_ emissions, as cellulolytic bacteria favor acetate production and thus produce more H_2_ that can be used in methanogenesis [29].

To formulate diets for ruminants or to anticipate *in vitro* and *in vivo* CH_4_ emissions related to feedstuffs, many researchers have derived prediction equations [27,42]. The majority of these proposed equations for CH_4_ prediction were created for the same categories of feedstuffs with very high precision [27]. In the present study, the equation using NDF and CP had an *R*^2^ = 0.76 (*p <* 0.002), whereas equations using CP and NDF individually had *R*^2^ = 0.72 (*p <* 0.001) and 0.45 (*p <* 0.01), respectively. The chemical composition (CP and NDF) of feedstuffs predicted the CH_4_ output in each category of feedstuffs, as demonstrated by the regression coefficient of the equations in this study. This is consistent with Santoso and Hariadi [40] and Singh et al. [27], who suggested that carbohydrate fractions (NDF and ADF) are stronger predictors of CH_4_ than feed components.

## Conclusion

The present study provides information on chemical constituents, *in vitro* digestibility, and estimated CH_4_ emissions of common feedstuffs used in the tropics, which could be used as a guideline to optimize diet formulation for ruminants to limit CH_4_ emissions from ruminants. The supplementation of Ipil-ipil to poor-quality roughages might be a way to reduce CH_4 _emissions and enhance animal production performance in the tropics. However, further studies on the CH_4_ emission profiles of common feedstuffs are required in addition to animal feeding trials based on the CH_4_ emission profiles of the ingredients.

## List of abbreviations

ADF, Acid detergent fiber; CH_4, _Methane; CP, Crude protein; dOM, Digestibility of organic matter; DM, Dry matter; GP, Gas production; HGT, Hohenheim Gas Test; ME, Metabolisable energy; NDF, Neutral detergent fiber; UMS, Urea molasses straw.
